# Hypermagnesemia in Clinical Practice

**DOI:** 10.3390/medicina59071190

**Published:** 2023-06-24

**Authors:** Aya Hasan Aal-Hamad, Abdullah M. Al-Alawi, Masoud Salim Kashoub, Henrik Falhammar

**Affiliations:** 1 Department of Emergency, Sultan Qaboos University Hospital, P.O. Box 141, Muscat 123, Oman; ayaalhamad96@gmail.com; 2 Department of Medicine, Sultan Qaboos University Hospital, P.O. Box 141, Muscat 123, Oman; dr.abdullahalalawi@gmail.com (A.M.A.-A.); masoud.kashoob@gmail.com (M.S.K.); 3 Internal Medicine Residency Training Program, Oman Medical Specialty Board, P.O. Box 1422, Al-Khoudh 132, Oman; 4 Department of Endocrinology, QB85, Karolinska University Hospital, 17176 Stockholm, Sweden; 5 Department of Molecular Medicine and Surgery, Karolinska Institute, 17177 Stockholm, Sweden

**Keywords:** magnesium homeostasis, hypomagnesemia, hypermagnesemia, electrolyte disturbance, clinical practice

## Abstract

Hypermagnesemia is a relatively uncommon but potentially life-threatening electrolyte disturbance characterized by elevated magnesium concentrations in the blood. Magnesium is a crucial mineral involved in various physiological functions, such as neuromuscular conduction, cardiac excitability, vasomotor tone, insulin metabolism, and muscular contraction. Hypomagnesemia is a prevalent electrolyte disturbance that can lead to several neuromuscular, cardiac, or nervous system disorders. Hypermagnesemia has been associated with adverse clinical outcomes, particularly in hospitalized patients. Prompt identification and management of hypermagnesemia are crucial to prevent complications, such as respiratory and cardiovascular negative outcomes, neuromuscular dysfunction, and coma. Preventing hypermagnesemia is crucial, particularly in high-risk populations, such as patients with impaired renal function or those receiving magnesium-containing medications or supplements. Clinical management of hypermagnesemia involves discontinuing magnesium-containing therapies, intravenous fluid therapy, or dialysis in severe cases. Furthermore, healthcare providers should monitor serum magnesium concentration in patients at risk of hypermagnesemia and promptly intervene if the concentration exceeds the normal range.

## 1. Introduction

Magnesium (Mg) is a vital mineral that functions as a cofactor in over 300 enzymatic reactions in the human body. It is essential for adenosine triphosphate (ATP) metabolism, DNA and RNA synthesis, and protein synthesis [1]. Moreover, it plays a critical role in regulating numerous physiological functions, including muscular contraction, blood pressure, insulin metabolism, cardiac excitability, vasomotor tone, nerve transmission, and neuromuscular conduction [1]. Hypomagnesemia is more common than hypermagnesemia and can lead to many neuromuscular, cardiac, or nervous disorders [2].

Previous studies indicate that hypomagnesemia is a prevalent electrolyte disturbance in clinical settings, particularly in patients admitted to the intensive care unit (ICU). Hypomagnesemia is associated with poor health outcomes, including prolonged length of hospital stay, increased mortality rate, and poor survival [1,3]. Unlike hypomagnesemia, hypermagnesemia is a less common condition and has not been as extensively studied in clinical practice. However, hypermagnesemia has been associated with poor health outcomes among hospitalized patients [4].

This narrative review summarizes the literature about hypermagnesemia, including its importance in clinical practice. This review seeks to improve patient outcomes and promote safe and effective clinical practice by extensively evaluating current research and clinical evidence.

## 2. Mg Transporters in the Human Body

**Figure 1:**
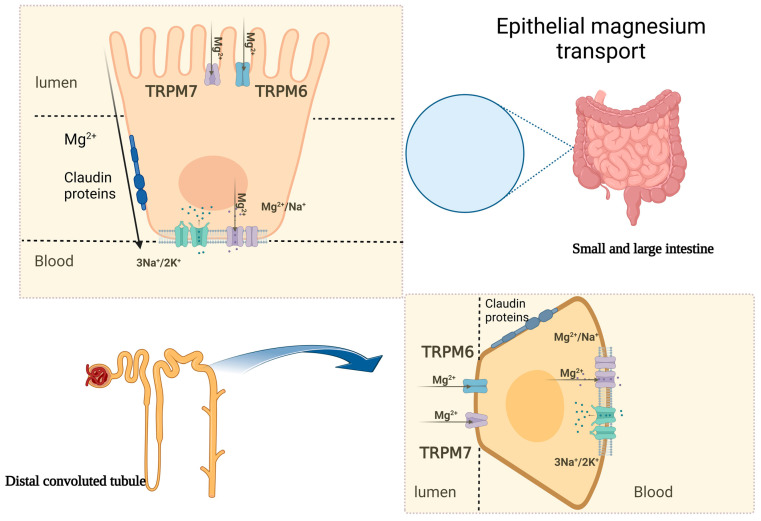
Epithelial magnesium transport in the intestine and kidney. The figure demonstrates the transcellular and paracellular mechanisms of Mg transportation. TRPM6/7: transient receptor potential melastatin cation channels 6 and 7.

The transportation of Mg involves eight cation transporters, which have been identified as transient receptor potential melastatin cation channels 6 and 7 (TRPM6, TRPM7), solute carrier family members 1 and 2 (SLC41A1, SLC41A2) channels, ancient conserved domain protein 2 (ACDP2), the mitochondrial RNA splicing 2 protein (Mrs2p), Mg transporter 1 (MagT1), human solute carrier family 41, and paracellin-1 [5]. TRPM7, located in various organs, including the heart, blood vessels, intestines, liver, lungs, brain, and spleen, is the most selective transport channel. Its existence is necessary for cell function survival [6]. TRPM6, on the other hand, regulates the body’s Mg concentration through the kidneys and intestines [7]. TRPM6 is mainly found in the colon and in the distal convoluted tubule (DCT), where such distribution strengthens the main role of regulating the Mg homeostasis in the human body (Figure 1) [8]. The relationship between TRPM6 expression and magnesium status was demonstrated in two mouse models. The findings revealed that dietary magnesium restriction and hypomagnesemia increase TRPM6 expression to enhance Mg absorption [9]. Mg efflux is carried out by several anti-porters and co-transporters, with the Na^+^/Mg^2+^ exchanger being considered the most important, and it is found on the smooth muscles of the heart and the blood vessels. Various factors might affect these exchangers, including vasopressin, angiotensin II, and insulin [5,10].

## 3. Mg Absorption in the Human Body

The majority of Mg absorption occurs in the small intestine via the paracellular pathway, which accounts for 80–90% of the total uptake [11]. The transcellular pathway also plays a minor role in Mg absorption in the colon [2,11]. The absorption of Mg electrolyte commences after 1 h of oral intake and reaches a stationary status—plateau—within 2–4 h, achieving 80% absorption after 6 h [11]. The paracellular pathway is the main process of Mg absorption in the small intestine. This is supported by the fact that Mg absorption in this area is strongly linked to the luminal Mg concentration, and TPRM6 channels are not expressed in the small intestines [11]. The paracellular transport is facilitated by high luminal Mg concentration and tight junction permeability regulated by claudins, allowing for simple diffusion due to the high concentration of Mg in the intestinal lumen and a lumen-positive trans-epithelial voltage [12]. TRPM6 and TRPM7, which belong to the TRPM family of cation channels, contribute to the active trans-cellular movement of the divalent cations from the large intestinal lumen into the cells (Figure 1) [11]. The rate of intestinal Mg absorption is not related to dietary intake but depends on the Mg status in the body. In situations where the dietary intake is scarce, the uptake of Mg is initiated actively via Mg-specific transporters in the colon, namely, TRPM6 and TRPM7 [13]. Pathological variants of the *TRPM6* gene have been linked to hypomagnesemia in patients with familial hypomagnesemia with secondary hypocalcemia [14].

## 4. Mg Storage and Renal Regulation

Almost all Mg in the body is stored, with less than 1% found in the serum and red blood cells. The main storage location for Mg is in the bone, with roughly one-third found on the bone surface, and it is related to the serum Mg concentration [15]. In the serum, Mg is found in the following three forms: two-thirds as ionized Mg, one-third as protein-bound Mg, and a minimal amount bound to anions. The kidneys play a vital role in maintaining Mg balance in the body, with 90–95% of filtered Mg being reabsorbed and only 3–5% excreted in the urine. The process of Mg reabsorption occurs along the nephron (Figure 1) [16]. Around 15–20% of reabsorption occurs in the proximal convoluted tubule, and the majority takes place in the thick ascending loop of Henle (TAL) (65–75%) [17]. The para-cellular Mg absorption in the TAL relies on the recognition of CLDN16 and CLDN19, and genetic variants in any of the aforementioned claudins can lead to familial hypomagnesemia, hypercalciuria and nephrocalcinosis (FHHNC) [17].

## 5. Determinants of Renal Mg Absorption

### 5.1. Hormonal

Several studies have shown that parathyroid hormone (PTH) affects the Mg absorption in the kidneys by acting on the loop of Henle and DCT. When PTH acts on the basolateral membrane of the cortical TAL at high concentration, it augments the sodium-chloride absorption. Subsequently, it enhances the trans-epithelial voltage, resulting in the paracellular absorption of Mg [17]. PTH also enhances the Mg absorption in the DCT in an otherwise not yet clearly understood mechanism. Although PTH activates renal Mg absorption, its effect on serum Mg concentration is not fully understood. Normal serum Mg concentration can be found in patients with primary hyperparathyroidism and hypoparathyroidism [16]. Similarly, calcitonin also influences the absorption of Mg in both the TAL and DCT. The epidermal growth factor is a hormone, which directly regulates the action of the TPRM6 channel in the DCT, and patients receiving anti-EGF receptor monoclonal antibodies such as cetuximab or panitumumab were at increased risk of hypomagnesemia with an overall incidence of 17% versus 3% in non-treated patients [18]. Furthermore, the transcription factor hepatocyte nuclear factor 1 homeobox B (HNF1β) also contributes to Mg reabsorption by stimulating the expression of FXYD2 protein. Hence, renal Mg wasting syndrome is found in patients with a pathological variant in the *FXYD2* gene [19].

### 5.2. Non-Hormonal

Several non-hormonal factors influence Mg absorption in the kidneys. Specifically, an elevation in luminal Mg concentration triggers Mg absorption by the kidneys, while an increase in peritubular concentration has the converse effect, leading to a decrease in Mg absorption. The calcium-sensing receptor (CaSR), present in the basolateral membrane of the TAL, plays a crucial role in this effect. The CaSR senses changes in extracellular calcium concentration and regulates the transport of other ions, including Mg [16]. This mechanism elucidates the reduced Mg excretion observed in instances of Mg depletion in the human body. Furthermore, serum acid-base status has been shown to impact Mg excretion by the kidneys. For example, metabolic acidosis has been linked to renal Mg wasting, while metabolic alkalosis has been associated with reduced renal Mg excretion [20].

## 6. The Role of Mg in Cellular Processes

At the cellular level, Mg plays a crucial role in the tertiary structure of both RNA and DNA. It helps stabilize the transfer RNA (t-RNA) structure and is essential for forming hydrogen bonds in DNA [21]. Additionally, Mg is a cofactor for several crucial enzymes in the human body, including topoisomerases, helicases, protein kinases, cyclases, glycolytic pathway enzymes, and ATP, which is vital for providing cellular energy [21,22].

## 7. Epidemiology of Hypermagnesemia

In clinical settings, the evaluation of serum total Mg concentration is the primary method used to assess Mg status, with the normal reference range typically being 0.7–1.0 mmol/L (1.7–2.4 mg/dL) [1,2,23]. The normal values may differ between laboratories, and some studies have employed slightly different ranges [23]. These discrepancies in normal values may partly account for the differences in reported Mg disorders prevalence among patients with similar characteristics [1].

Unlike hypomagnesemia, few studies assessed the prevalence of hypermagnesemia in various health settings [24,25,26,27]. In a population-based prevalence study evaluating the prevalence of Mg concentrations in an urban general population (*n* = 1558), the overall prevalence of hypermagnesemia was 3.0% [26]. Pregnant women with eclampsia are considered to be a high-risk group due to the need for high doses of intravenous Mg to prevent eclamptic seizures [28]. However, Mg intoxication incidence was in a large systematic review involving 9556 women with Mg use due to pre-eclampsia, only 1.3–1.6% [27].

In a hospital setting, the prevalence of hypermagnesemia ranges from 5.7% to 9.3% [24,29]. The most extreme cases of elevated serum Mg concentration recorded were in a premature infant at 33 weeks gestation with a concentration of 18 mmol/L and in a 78-year-old woman who ingested water from the Dead Sea with a concentration of 13.4 mmol/L [24,25]. It has been estimated that around 10% to 15% of hospitalized patients with renal failure develop hypermagnesemia due to reduced renal excretion of Mg [30]. Hypermagnesemia was associated with poor health outcomes, including increased in-hospital mortality and 1-year mortality among hospitalized patients [4,31,32]. Moreover, hypermagnesemia strongly predicts the in-hospital mortality rate of acute myocardial infarction [33]. This may be due to Mg ions competing with the calcium ions for activation and deactivation sites located on the type II isoform ryanodine receptor channels in cardiac myocytes, damaging cardiac contraction, and relaxation [34]. Additionally, Mg can also impair the release of acetylcholine, leading to motor end-plate sensitivity depression and inducing arrhythmia, myocardial depression, and vasodilation [35].

Moreover, hypermagnesemia appears to be an indicator of disease severity among patients hospitalized with SARS-CoV-2, and hypermagnesemia was associated with prolonged hospitalization, higher rates of ICU admission, greater need for mechanical ventilation, and mortality [36].

## 8. Assessment of Mg Status

The commonly used method is measuring total serum magnesium concentration, although it may not provide the most reliable evaluation due to potential influences from serum protein concentrations. Recent advancements in ion-selective electrodes have enabled the measurement of ionized magnesium concentration, but standardization is needed. Red cell magnesium concentration does not correlate well with overall magnesium status while assessing magnesium content in mononuclear cells is technically challenging. Muscle magnesium content assessment through invasive procedures has shown promise in predicting cardiac magnesium levels [1,35].

A 24-h urine excretion of magnesium can reflect intestinal absorption and identify renal magnesium wasting. The magnesium tolerance test accurately determines magnesium retention after intravenous administration, but its utility is limited in patients with renal magnesium loss. Intracellular free magnesium concentration can be measured using fluorescent probes or nuclear magnetic resonance [1,37].

Magnesium balance studies, isotopic analysis, hair and tooth analysis, and enzyme activation studies have been explored, but they are less reliable than serum or red cell magnesium concentration. Overall, a combination of tests, such as measuring total serum magnesium and employing the magnesium tolerance test, currently provides the most accessible assessment of magnesium status. With advancements in technology, ionized magnesium measurement may become more widely available and reliable in the future [1,38].

## 9. Causes of Hypermagnesemia

### 9.1. Reduced Renal Excretion

Patients with either acute kidney injury or chronic kidney disease (CKD) are at increased risk of hypermagnesemia due to the importance of the renal system for Mg excretion, and 10–15% of hospitalized patients with kidney injury may develop hypermagnesemia [33]. Moreover, in rare circumstances, several endocrinological conditions might cause marked rises in serum Mg concentrations, such as hyperparathyroidism, adrenal insufficiency, and hypothyroidism, by increasing Mg renal reabsorption [39,40]. Hyperparathyroidism and calcium metabolism disturbance can result in hypermagnesemia through an increased calcium-induced Mg absorption in the tubule [41]. Familial hypocalciuric hypercalcemia (FHH) is a rare autosomal dominant condition that occurs due to a variant in the calcium-sensing receptor gene (*CaSR*). The CaSR presents in all the kidney segments and prominently in the basolateral side of TAL, controlling the sodium chloride and divalent cation, such as Mg and calcium, transportation both transcellularly and paracellularly by enhancing various channels such as NKCC2 and ROMK [42]. As a result of *CaSR* gene variations, the aforementioned channels (NKCC2 and ROMK) get over-activated and create a positive activity in the lumen, which encourages the action of the paracellin, which induces the reabsorption of the Mg and calcium transcellularly and paracellularly. In hypothyroidism, it is suggested that Mg excretion is impaired due to a drop in renal blood flow and filtration rate [43,44]. Increased sodium-potassium ATPase activity in settings of hypothyroidism has been reported, which results in a high electrochemical gradient leading to reabsorption of Mg. The latter could explain the association between hyperkaliemia and hypermagnesemia [44,45,46,47]. Finally, certain drugs that act on renal endothelial vessels and the angiotensin system might cause hypermagnesemia, such as lithium, angiotensin-converting enzyme inhibitors, and non-steroidal anti-inflammatory drugs (Table 1) [41].

### 9.2. Increased Intake of Mg

Hypermagnesemia might develop in individuals despite normal renal functions, especially in elderly patients with certain bowel conditions that enhance the absorption or reduce gut motility, including inflammatory bowel diseases and constipation [48]. Similarly, anticholinergics medications or laxatives might result in high serum Mg concentrations, primarily in settings of pre-existing bowel pathology [49]. Medications containing Mg can elevate serum Mg concentrations if taken continuously, particularly when renal function is impaired. Amaguchi and colleagues reported a case of symptomatic hypermagnesemia secondary to Mg supplements in an elderly with underlying constipation [48]. In order to evaluate the risk of hypermagnesemia in patients using magnesium oxide tablets, a retrospective study was conducted involving 2176 individuals who took daily magnesium oxide for laxative purposes. The study indicated a correlation between hypermagnesemia and CKD grade 4 and higher dosages of magnesium oxide. Moreover, elevated serum Mg concentration was associated with magnesium oxide dosage exceeding 1000 mg/day, CKD grade 4, and the concurrent use of stimulant laxatives [50]. Milk alkali syndrome may also cause hypermagnesemia, as reported already in 1936 by Cope, where patients developed toxic symptoms of hypercalcemia, hyperphosphatemia, hypermagnesemia, and azotemia secondary to calcium carbonate-containing alkali therapy [51,52]. In patients undergoing dialysis, increased dialysate Mg can also cause symptomatic hypermagnesemia [53]. Moreover, a case report demonstrated hypermagnesemia in a patient with post-urethral irrigation with hemiacidrin, which is utilized in the nephrolithiasis process [54]. Moreover, excessive infusion of Mg sulfate during the management of eclampsia is a well-known cause of hypermagnesemia, which can be fatal [41,55]. Hypermagnesemia resulting solely from dietary intake is not reported thus far, as the kidneys effectively eliminate excess magnesium through urine. However, patients with CKD are more susceptible to developing hypermagnesemia due to impaired renal excretion. Hence, educating these patients about minimizing their consumption of magnesium-rich foods, such as seeds, nuts (such as almonds and cashews), black beans, brown rice, bananas, and broccoli, is crucial [56].

### 9.3. Mg Leak to the Extracellular Fluid

Mg is an essential intracellular cation. Consequently, in scenarios where hemolysis occurs due to various causes, including tumor lysis syndrome, there is a potential risk of developing hypermagnesemia [57]. Other causes which can present with hypermagnesemia through the extracellular shifts include rhabdomyolysis and metabolic acidosis, including diabetic ketoacidosis [58]. Metabolic acidosis causes urinary Mg wasting as a compensatory mechanism for the rapid rise in serum Mg [59]. Hence, chronic low-grade metabolic acidosis in humans eating Western diets may contribute to decreased Mg status [60].

## 10. Clinical Manifestations of Hypermagnesemia

Hypermagnesemia might go unnoticed initially due to the non-specificity of the symptoms and wide variability. Generally, hypermagnesemia is well tolerated, and concentrations between 1.05 and 2.2 mmol/L (2.55–5.35 mg/dL) can be completely asymptomatic [46]. Concentrations between 2.2 and 3.5 mmol/L (5.35–8.5 mg/dL) cause non-specific symptoms such as nausea, dizziness, weakness, and confusion [37]. As it increases above 3.5 mmol/L (8.5 mg/dL), more neurological manifestations occur, such as worsening confusion, drowsiness, and depressed reflexes, as well as headache, flushing, urinary complications due to bladder paralysis, and gastrointestinal symptoms. Patients may also experience blurred vision due to impaired eye accommodation and convergence and a mild decrease in blood pressure (Table 2) [21,61].

More serious symptoms and signs might develop at concentrations above 6.5 mmol/L (15.8 mg/dL), such as paralytic ileus, muscle paralysis, bradypnea, and hypotension. Hypermagnesemia can also induce electrocardiogram (ECG) changes, including sinus bradycardia, prolonged PR and QRS interval, and atrioventricular block [37,62]. Other reported rare ECG changes include ST elevation and prominent T wave [63,64].

**Figure 2:**
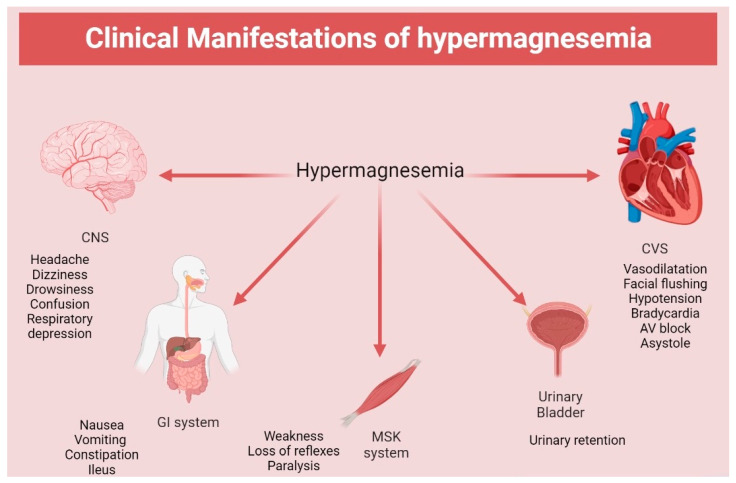
Clinical manifestation of hypermagnesemia. CNS: central nervous system; CVS: cardiovascular system; GI: gastrointestinal system; MSK: musculoskeletal.

The serum Mg concentration exceeding 8.7 mmol/L (21.1 mg/dL) might lead to coma and cardiac arrest [21]. There are some cases in which patients presented with choreiform movements and seizures when hypermagnesemia was combined with hypocalcemia (Figure 2) [41].

## 11. Clinical Assessment of Hypermagnesemia

When managing a patient with hypermagnesemia, a comprehensive assessment should be conducted to evaluate the severity of the condition and identify its underlying cause. This includes obtaining a detailed medication history, as certain drugs have been associated with hypermagnesemia [65,66]. Additionally, it is important to explore the patient’s medical history, particularly for constipation or inflammatory bowel disease, which may increase the risk of hypermagnesemia [37].

Laboratory testing is a critical evaluation component and should include measuring serum Mg concentration and assessing renal function with tests such as glomerular filtration rate (GFR), creatinine, blood urea nitrogen (BUN), glucose, and urine specific gravity [66]. Arterial blood gas analysis may help detect the presence of acidosis, and a thyroid function test may be conducted to rule out hypothyroidism as a possible cause. Since hyperkalemia and hypercalcemia often coexist with hypermagnesemia, potassium, calcium, and phosphate concentrations should also be measured [46,65]. Patients with moderate to severe hypermagnesemia and those with any symptoms should be closely monitored for cardiac abnormalities. Therefore, clinicians should maintain a high level of vigilance and conduct regular cardiac monitoring to prevent any adverse events [37].

## 12. Management of Hypermagnesemia

The treatment approach for hypermagnesemia varies depending on the severity of the electrolyte imbalance. Patients with mild hypermagnesemia and optimal renal function may not require any treatment except for discontinuing the source of Mg and careful monitoring. However, patients with more severe symptoms, such as neurological manifestations, ECG changes, hypotension, and reduced respiratory rate, require immediate treatment [37].

The initial treatment for severe hypermagnesemia involves the intravenous administration of calcium gluconate or chloride to counteract the effects of Mg on the neuromuscular junction and the heart. A typical dosage is 1 g given over 2–5 min, followed by an infusion of 150–100 mg of calcium over 5–10 min. Normal saline may also be given at a 150–200 mL/h rate to augment renal excretion of Mg [37].

**Figure 3:**
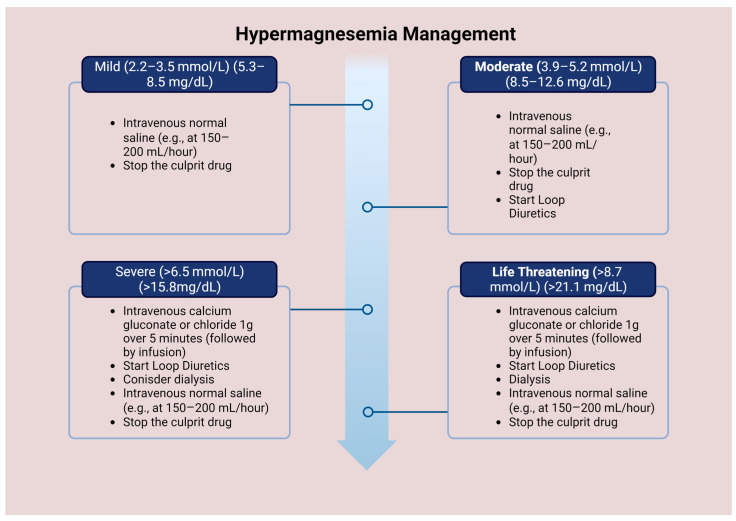
Management of hypermagnesemia according to Mg concentration.

In critical cases of hypermagnesemia, it is crucial to increase Mg excretion through the kidneys. This can be accomplished using two methods. The first approach involves using loop diuretics, such as furosemide, at a dosage of 1 mg/kg. Intravenous fluid saline infusion is necessary to prevent electrolyte imbalances, including hypokalemia and metabolic alkalosis [65]. The second approach is hemodialysis, which is particularly effective for patients with renal failure. This method can remove almost 50% of serum Mg after a 3–4 h treatment. However, hemodialysis can also cause hypocalcemia due to increased calcium excretion, which may worsen the signs and symptoms of hypermagnesemia (Figure 3) [67].

## 13. Conclusions

Diagnosing hypermagnesemia presents a challenge due to its rarity and the absence of routine monitoring of Mg concentrations. Moreover, the signs and symptoms are non-specific, often leading to a delayed diagnosis. The prognosis of hypermagnesemia is contingent on the extent of the electrolyte imbalance. Favorable outcomes are expected in patients with mild hypermagnesemia and no precipitating factors, while those with severe hypermagnesemia and acute presentations are at an increased risk of mortality. Caution should also be exercised in using laxatives or Mg-containing antacids to prevent hypermagnesemia. In summary, timely identification and management of hypermagnesemia are critical to prevent potentially fatal complications, especially in patients with renal impairment.

## Figures and Tables

**Table 1:** Causes of hypermagnesemia.

Category	Sub-Category	Causes	Mechanisms
Reduced Renal Excretion	Kidney Disease	Acute kidney injury Chronic kidney disease	Reduced ability of the kidneys to excrete Mg
	Endocrinological Conditions	Hyperparathyroidism Adrenal insufficiency Hypothyroidism	Abnormalities in calcium metabolism or renal blood flow/filtration rate
	Certain Drugs	Lithium, angiotensin-converting enzyme inhibitors, non-steroidal anti-inflammatory drugs	Alteration of renal endothelial vessels and angiotensin system
Increased Intake of Mg	Bowel Conditions	Elderly patients with bowel conditions	Enhanced absorption or reduced gut motility
	Medications	Anticholinergics, laxatives, and medications containing Mg	Increased intake or absorption of Mg
	Other Causes	Milk alkali syndrome, increased dialysate Mg, post-urethral irrigation with hemiacidrin, excessive infusion of Mg sulfate	Various mechanisms
Mg Leak to Extracellular Fluid	Hemolysis	Tumor lysis syndrome	Movement of Mg from intracellular to extracellular space
	Metabolic Acidosis	Diabetic ketoacidosis	Movement of Mg from intracellular to extracellular space
	Other Causes	Chronic low-grade metabolic acidosis	Various mechanisms

**Table 2:** Symptoms of hypermagnesemia and the corresponding magnesium concentrations.

Symptoms	Magnesium Concentration
Non-specific symptoms, wide variability	<2.2 mmol/L (<5.35 mg/dL)
Nausea, dizziness, weakness, confusion	2.2–3.5 mmol/L (5.35–8.5 mg/dL)
Worsening confusion, drowsiness, depressed reflexes, headache, flushing, urinary complications, gastrointestinal symptoms, blurred vision	>3.5 mmol/L (>8.5 mg/dL)
Mild decrease in blood pressure	>3.5 mmol/L (>8.5 mg/dL)

## Data Availability

Not applicable.
